# Ultra-Fast Construction of Novel S-Scheme CuBi_2_O_4_/CuO Heterojunction for Selectively Photocatalytic CO_2_ Conversion to CO

**DOI:** 10.3390/nano12183247

**Published:** 2022-09-19

**Authors:** Weina Shi, Xiu Qiao, Jichao Wang, Miao Zhao, Hongling Ge, Jingjing Ma, Shanqin Liu, Wanqing Zhang

**Affiliations:** 1 School of Chemistry and Materials Engineering, Xinxiang University, Xinxiang 453000, China; 2 College of Chemistry and Chemical Engineering, Henan Institute of Science and Technology, Xinxiang 453000, China

**Keywords:** CO_2_ reduction, photocatalyst, selectivity, CuBi_2_O_4_/Cu_2_O, S-scheme

## Abstract

Herein, step-scheme (S-scheme) CuBi_2_O_4_/CuO (CBO/CuO) composite films were successfully synthesized on glass substrates by the ultra-fast spraying-calcination method. The photocatalytic activities of the obtained materials for CO_2_ reduction in the presence of H_2_O vapor were evaluated under visible light irradiation (λ > 400 nm). Benefiting from the construction of S-scheme heterojunction, the CO, CH_4_ and O_2_ yields of the optimal CBO/CuO composite reached 1599.1, 5.1 and 682.2 μmol/m^2^ after irradiation for 9 h, and the selectivity of the CO product was notably enhanced from below 18.5% to above 98.5% compared with those of the bare samples. In the sixth cycling experiment, the yields of main products decreased by less than 15%, and a high CO selectivity was still kept. The enhanced photocatalytic performance of CO_2_ reduction was attributed to the efficient separation of photogenerated charge carriers. Based on the photocatalytic activity, band structure and in situ-XPS results, the S-scheme charge transfer mechanism was conformed. The study provides an insight into the design of S-scheme photocatalysts for selective CO_2_ conversion.

## 1. Introduction

Photocatalytic reduction of CO_2_ into valuable chemicals utilizing H_2_O and solar energy is considered to be a promising strategy for addressing energy shortage and greenhouse effect issues. On the basis of that, substantial efforts have been devoted to developing highly efficient photocatalysts, and copper-containing oxide photocatalysts are particularly relevant for CO_2_ reduction, including CuO, Cu_2_O, CuBi_2_O_4_, CuFe_2_O_4_, CuFeO_2_, CuV_3_O_4_, Cu_3_Nb_2_O_8_, CuGaO_2_, and so on [1]. Among the above encouraging photocatalysts, CuBi_2_O_4_ is an attractive candidate in terms of its suitable band gap (1.5–2.0 eV) and relatively negative conduction band edge position [1]. However, the major hurdle for CuBi_2_O_4_ lies in its valance band potential, which is more positive than the H_2_O/O_2_ potential, indicating its main application in the field of photoelectrocatalysis rather than photocatalytic CO_2_ reduction with H_2_O [2]. Hence, it is necessary to devise CuBi_2_O_4_-based composite catalysts with a high conversion efficiency and controllable selectivity for photocatalytic CO_2_ reduction.

The formation of a heterojunction structure by coupling CuBi_2_O_4_ with other semiconductors is a feasible strategy for photocatalyst modification [3]. Particularly, the construction of a S-scheme heterojunction can preserve strong redox abilities and achieve an efficient separation of charge carriers in comparison with the traditional type II heterojunction, enhancing photocatalytic activity and product selectivity for CO_2_ reduction [4,5,6,7]. For instance, CuBi_2_O_4_-based S-scheme systems such as BiOCl/CuBi_2_O_4_ [8], CuBi_2_O_4_/BiOBr [9], CuBi_2_O_4_/CoV_2_O_6_ [10] and CuBi_2_O_4_/Bi_4_O_5_I_2_ [11] have been reported to boost photocatalytic performances. Our previous work demonstrated that the synthesized S-scheme CuBi_2_O_4_/Bi_2_O_3_ heterojunction exhibited enhanced CO and CH_4_ yields for CO_2_ photoreduction in water vapor [2]. We further reported the construction of a S-scheme WO_3_/CuBi_2_O_4_ photocatalyst for a visible-light-driven CO_2_ reduction with a good photocatalytic activity and stability [12]. As a typical transition metal oxide, CuO has been demonstrated to be feasible for heterojunction construction due to its narrow band gap (1.4−1.8 eV) and suitable band edge position [13], and heterostructures including CuO/TiO_2_ [14], CuO/ZnO [15], WO_3_/CuO [16,17], CuO/BiOCl [18], CuO/g-C_3_N_4_ [19] and Nb_2_O_5_/CuO [20] have been adopted for efficient photocatalytic CO_2_ photoreduction. For instance, Nogueira et al. reported that compositing Nb_2_O_5_ with increased amounts of CuO led to a higher selectivity for CH_4_ production [20]. Therefore, in view of the above analysis, it is of significance to rationally construct the heterojunction catalyst by combining CuO and CuBi_2_O_4_. Recently, CuO/CuBi_2_O_4_ heterojunction has been studied for photoelectrocatalysis [21,22,23], photodegradation [24] and electrochemical detection [25]. However, to date, few studies have been conducted to construct CuO/CuBi_2_O_4_ heterojunction for photocatalytic CO_2_ reduction with H_2_O vapor, a system in which the limited solubility of CO_2_ in the solvent or weak CO_2_ adsorption ability will be overcome.

In this work, a series of CuO/CuBi_2_O_4_ heterojunction composites with different molar ratios were synthesized onto glass substrates by ultra-fast spray pyrolysis followed by annealing treatment. The heterojunction catalysts were investigated for photocatalytic CO_2_ reduction with H_2_O vapor. The CuO/CuBi_2_O_4_ composites exhibited a superior photocatalytic performance than those of the single CuO and CuBi_2_O_4_, and the enhanced ratio of CuBi_2_O_4_ in the heterojunction benefited the improved product selectivity of CO_2_ photoreduction into CO. Based on the experimental and theoretical results, the S-scheme charge transfer mechanism was proposed and discussed in detail.

## 2. Materials and Methods

### 2.1. Materials Synthesis

In the typical synthesis of the CuBi_2_O_4_/CuO sample, 2.6180 g of Cu(NO_3_)_2_·3H_2_O was firstly dissolved into 20 mL of ethanol, and 2.4250 g of Bi(NO_3_)_3_·5H_2_O was dissolved into 4 mL of nitric acid with the subsequent addition of deionized water (16 mL). The precursor solution for spraying was obtained by mixing the above two solutions. The glass substrate (2.1 cm × 2.3 cm) was pre-cleaned in H_2_O_2_ solution and irradiated by UV light for 30 min, followed by immobilization onto the heating stage under 280 °C, and then the precursor solution was sprayed on the above substrate with 0.3 MPa N_2_ pressure. The obtained precursor film was heated at 480 °C for 2 h in air. Finally, the CuBi_2_O_4_/CuO composite was obtained and named as 30CBO/CuO (30 represented the ideal molar ratio between CuBi_2_O_4_ and CuO). In addition, the other CBO/CuO composites with different ratios of two components were similarly synthesized by spraying the precursor solutions with variations in the contents of Cu(NO_3_)_2_·3H_2_O and Bi(NO_3_)_3_·5H_2_O. For comparison, the bare CuO and CuBi_2_O_4_ samples were synthesized based on the stoichiometric mole ratios of Cu and Bi in the precursor solutions. Simultaneously, the 30CBO/CuO-m sample was obtained by mechanically mixing the bare CuO and CuBi_2_O_4_ nanoparticles with a molar ratio of 0.3.

### 2.2. Characterization

The crystal structures of the as-prepared samples were studied by X-ray diffraction (XRD, BRUKER D8 Advance). The morphology and structure were inspected with scanning electron microscopy (SEM, FEI Quanta 250 FEG) and transmission electron microscopy (TEM, FEI Talos F200X). The chemical states were investigated by X-ray photoelectron spectroscopy (XPS, Escalab XI+, Thermofisher Scientific, Santa Clara, CA, USA). The optical properties of the obtained photocatalysts were obtained on a UV-vis spectrophotometer (Cary 5000, Agilent, Santa Clara, CA, USA). The photocurrent responses and electrochemical impedance spectroscopy were measured by an electrochemical workstation (CHI660E, CH Instruments Ins., Shanghai, China).

### 2.3. Photocatalytic Performance for CO_2_ Reduction

The photocatalytic performance for CO_2_ reduction with H_2_O vapor was measured in the reactor with a top quartz window. A 300 W Xenon arc lamp (CEL-HXF300, Beijing China Education Au-light Co., Ltd., Beijing, China) with an UV cutoff filter (λ > 400 nm) was utilized as the light source. The glass substrate with the composite catalyst was placed inside the stainless steel cylindrical vessel reactor (CEL-GPRT100, Beijing China Education Au-light Co., Ltd., China). Prior to light irradiation, the reaction setup was purged with high purity CO_2_ gas (99.999%) several times. The compressed high-purity CO_2_ gas was passed through a water bubbler to generate a mixture of CO_2_ and H_2_O vapor. After illumination, the gaseous products were quantitatively identified for off-line analysis using the gas chromatograph (GC-7920, Beijing China Education Au-light Co., Ltd., China) equipped with a flame ionized detector (FID) and GC-MS (Agilent 7890A-5975C) with a thermal conductivity detector (TCD), and the equipped columns were TDX-01 and Porapak-Q, respectively. Blank tests were conducted in the absence of photocatalysts and in the dark with catalysts. No products were detected, indicating that the presence of both visible-light irradiation and the photocatalyst were indispensable for the photocatalytic reduction of CO_2_ with H_2_O vapor. The product selectivity (*S*) for CO_2_ reduction was calculated with the following equations (Equations (1) and (2)):

*S*_CO_ (%) = 2 × *N*_CO_/(2 × *N*_CO_ + 8 × *N*_CH_4__) × 100 (1)

*S*_CH_4__ (%) = 2 × *N*_CH_4__/(2 × *N*_CO_ + 8 × *N*_CH_4__) × 100 (2)

*N*_CO_ and *N*_CH_4__ represented the yield of detected CO and CH_4_ molecules, respectively. To evaluate the stability, the 30CBO/CuO model sample was refreshed by washing with deionized water, and its photocatalytic performance was reevaluated in the aforementioned conditions.

## 3. Results and Discussion

### 3.1. Structure, Composition and Morphology

**Figure 1:**
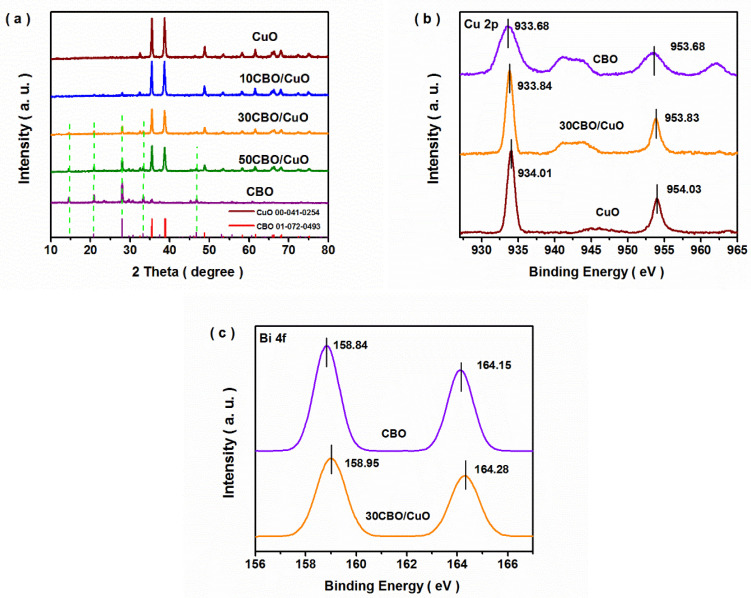
XRD patterns (**a**) and XPS spectra ((**b**) Cu 2p and (**c**) Bi 4f) of the as-prepared samples.

Figure 1a shows the XRD patterns of the as-prepared samples. Several diffraction peaks were distinctly discovered at 35.5, 38.7, 48.7 and 61.5°, which corresponded to the (−1 1 1), (1 1 1), (−2 0 2) and (−1 1 3) crystal faces of monoclinic CuO (PDF card No. 00-041-0254). Meanwhile, the diffraction peaks of the CuBi_2_O_4_ sample matched perfectly with those of tetragonal CuBi_2_O_4_ (PDF card No. 01-072-0493). Simultaneously, the peak intensity of CuBi_2_O_4_ in the CBO/CuO composites, which was labeled by a green dotted line in Figure 1a, gradually increased with increasing molar ratios of CuBi_2_O_4_. Except for the diffraction peaks of CuBi_2_O_4_ and CuO, no other peaks were detected in the composites, which indicated that the CBO/CuO composite was composed of monoclinic CuO and tetragonal CuBi_2_O_4_. XPS measurements were further conducted to explore the composition of the obtained samples. As shown in Figure S1a, the Cu, Bi, O and C elements existed in the CBO and composite samples, while Cu, O and C elements appeared in the CuO sample. The C element was attributed to the adsorbed CO_2_ molecules on the surface. In the high-resolution Cu 2 p XPS spectra (Figure 1b) of the CBO/CuO composite, two peaks were observed at binding energies of 933.84 eV for Cu(II) 2 p 3/2 and 953.83 eV for Cu(II) 2 p 1/2 [26,27]. Furthermore, the conspicuous peaks at binding energies of 158.84 and 164.15 eV in the Bi 4f spectra (Figure 1c) were ascribed to the Bi(III) state of CuBi_2_O_4_ [28,29]. It was notable that a slight shift of the characteristic peak position appeared for the 30CBO/CuO composite when compared with those of the bare CuBi_2_O_4_ and CuO samples, which was caused by the formation of a heterojunction between the two components. The O 1 s spectrum of CBO/CuO is shown in Figure S1b, and the two peaks at 530.1 and 529.6 eV were ascribed to lattice oxygen of CuO and CuBi_2_O_4_ materials. Other peaks at 531.2 and 532.5 eV were assigned to intrinsic oxygen defects and surface adsorbed oxygen, respectively [20,24,30].

**Figure 2:**
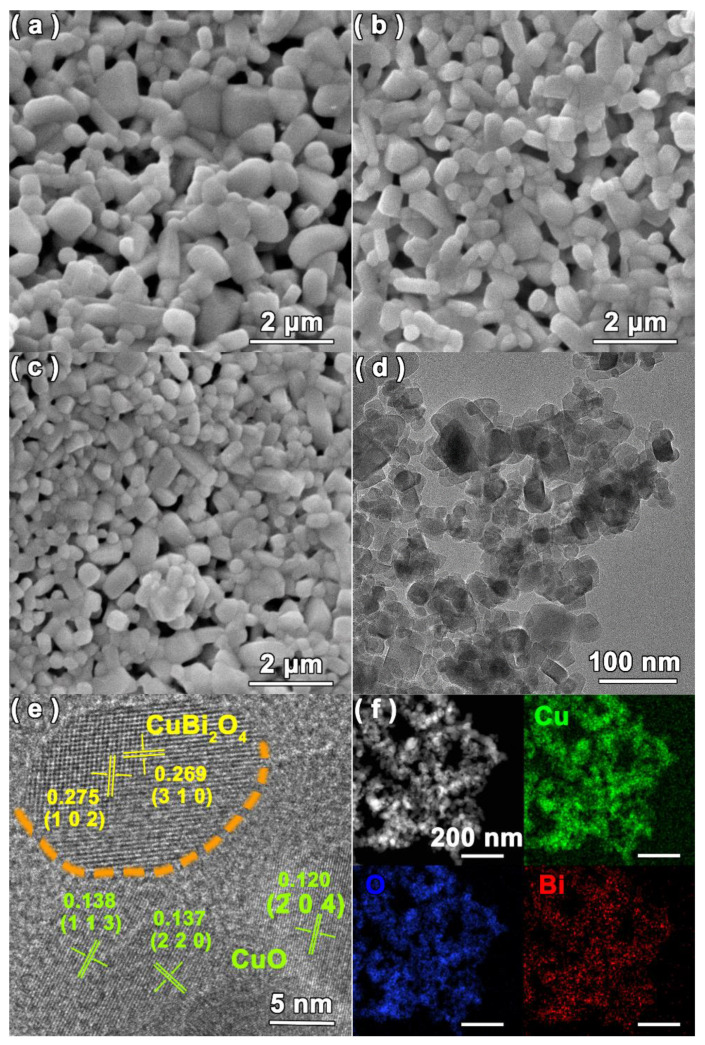
SEM images of (**a**) CuBi_2_O_4_, (**b**) CuO and (**c**) 30CBO/CuO samples; (**d**) TEM image, (**e**) HRTEM image and (**f**) EDX mapping images of the 30CBO/CuO sample.

The morphologies of the as-prepared CuBi_2_O_4_, CuO and 30CBO/CuO samples were visualized via SEM and TEM characterizations. The SEM images in Figure 2a–c demonstrated that all the samples exhibited the typical particle morphology. Concurrently, a lot of voids were formed due to piles of particles, leading to large exposed surfaces. TEM measurements were further conducted to determine the accurate sizes of CuO and CuBi_2_O_4_ particles, which were obtained from the substrates by ultrasound methods. As shown in Figure S2, the size distribution of CuO and CuBi_2_O_4_ nanoparticles basically obeyed the logical normal distribution, and their average particle sizes were about 47.7 and 26.6 nm, respectively. The TEM image of 30CBO/CuO in Figure 2d shows two groups of particles with different sizes. In the HRTEM image (Figure 2e), the distinct lattice spacings of 0.275 and 0.268 nm were indexed to the (1 0 2) and (3 1 0) crystal faces of tetragonal CuBi_2_O_4_, and lattice spacings of 0.138, 0.137 and 0.120 nm were also discovered, which were assigned to the (1 1 3), (2 2 0) and (2 0 4) planes of monoclinic CuO. Additionally, the EDX mapping images in Figure 2f showed that the Cu, Bi and O elements were uniformly distributed. The above SEM and TEM observations, combined with the XRD and XPS results, revealed that both monoclinic-phase CuO and tetragonal-phase CuBi_2_O_4_ were present and fused at the interface.

**Figure 3:**
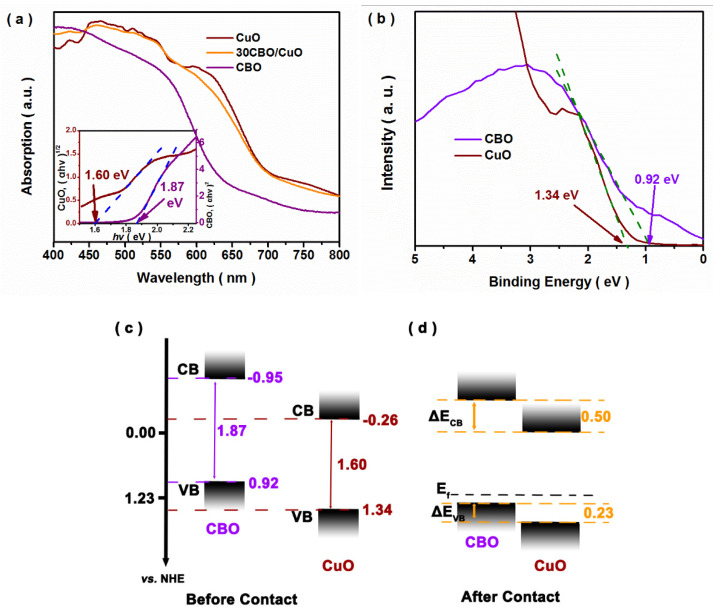
(**a**) DRS and (**b**) VB-XPS spectra of the as-prepared samples; and (**c**,**d**) schematic diagrams of the band structure for CBO/CuO heterojunction.

To further explore the band structure of heterojunction, the DRS and VB-XPS results were gained and are shown in Figure 3. As shown in Figure 3a, the bare and composite samples showed a strong absorption in the visible light region, indicating the feasibility of a visible-light utilization for photocatalytic CO_2_ reduction. The band gaps (*E*_g_) of the samples were estimated by the plots of (*αhν*)^n^ versus photo energy (*hν*), considering that CuBi_2_O_4_ and CuO exhibited band-to-band excitations involving direct and indirect transitions, respectively [20,31]. The *E*_g_ values of CuBi_2_O_4_ and CuO were respectively calculated to be 1.87 and 1.60 eV. According to the conventional method, the valence band (VB) positions of the CuBi_2_O_4_ and CuO samples (Figure 3b) were determined to be about 0.92 and 1.34 eV, respectively. Hence, their conduction band (CB) positions were calculated to be −0.95 and −0.26 eV, respectively. With the formation of the heterojunction, the alignment of the Fermi levels was assumed at the interfacial phases of the CBO/CuO composite. Accompanied by the movement of the Fermi levels, the CB and VB edge positions for both CuBi_2_O_4_ and CuO shifted. In this case, the band offsets of the CBO/CuO heterojunction can be calculated using the XPS core-level alignment method, according to the following equation [24,32]:

Δ*E*_VB_ = (*E*_Bi4f7/2,CBO_ − *E*_VB,CBO_) − (*E*_Cu2p3/2,CuO_ − *E*_VB,CuO_) − (*E*_Bi4f7/2,CBO/CuO_ − *E*_Cu2p3/2,CBO/CuO_) (3)

Δ*E*_CB_ = Δ*E*_VB_ − *E*_gap,CBO_ + *E*_gap,CuO_ (4)

where *E_x,y_* was denoted as the binding energy of the core level “*x*” or VB for the sample “*y*”, and *E*_gap, y_ referred to the values calculated from DRS. According to the above method and relevant data (Table S1), the Δ*E*_VB_ and Δ*E*_CB_ for the CBO/CuO composite were calculated to be about 0.23 and 0.15 eV. The corresponding band structure diagrams (Figure 3c,d) were schemed, and a staggered band alignment heterostructure was constructed.

### 3.2. Photocatalytic Performance of CO_2_ Reduction

**Figure 4:**
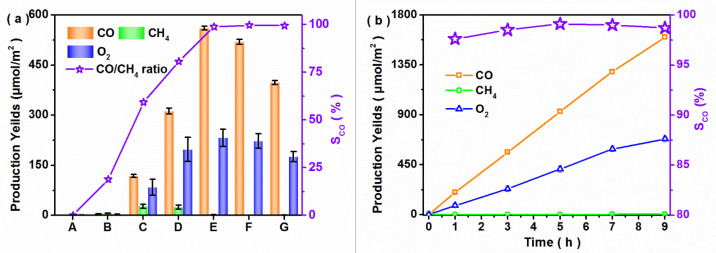
Photocatalytic activity for different samples under visible-light illumination ((**a**) A. CBO, B. CuO, C. 10CBO/CuO, D. 20CBO/CuO, E. 30CBO/CuO, F. 40 CBO/CuO, G. 50CBO/CuO. (**b**) 30CBO/CuO as catalyst).

The photocatalytic performances for CO_2_ reduction were investigated under the mixed atmosphere of CO_2_ and H_2_O vapor. Figure 4a shows the production yields after 3 h of visible-light irradiation (>400 nm). The photocatalytic products for the bare CuO catalyst included CO, CH_4_ and O_2_, while there were no detectable products for bare CuBi_2_O_4_. The CO, CH_4_ and O_2_ yields of 30CBO/CuO were the highest among the CBO/CuO composites with different ratios. Simultaneously, the CO selectivity was obviously enhanced with the increase of the CuBi_2_O_4_ content in the composite, reaching about 98% for the 30CBO/CuO catalyst. Compared with the photocatalytic activity of 30CBO/CuO-m (Figure S3), the enhanced yields were caused by the formation of a heterojunction in the 30CBO/CuO composite. CO, CH_4_ and O_2_ yields respectively reached 561.4, 2.1 and 232.2 μmol/m^2^ after 3 h of visible-light illumination. The yields of the main products rapidly and regularly increased (Figure 4b) when prolonging the illumination time. After 9 h of visible-light illumination, the rate of CO and O_2_ yields were still maintained at 177.7 and 75.8 μmol/m^2^/h, respectively, and the CO selectivity was kept above 98.5%.

**Figure 5:**
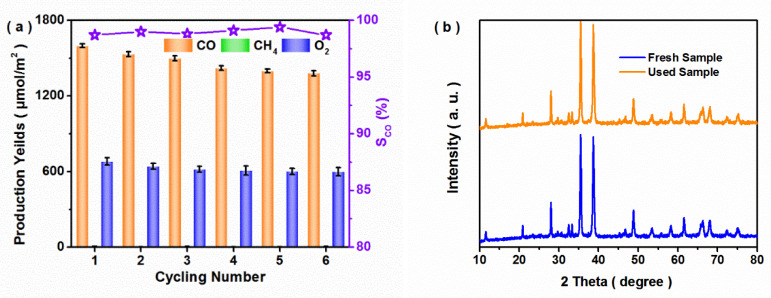

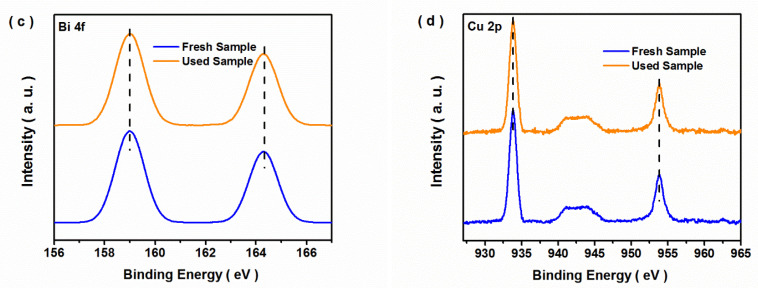
Photocatalytic activity (**a**) of 30CBO/CuO in the cycling experiment; (**b**) XRD pattern, (**c**) Bi 4f and (**d**) Cu 2p XPS spectra of the fresh and used 30CBO/CuO samples after the sixth cycling experiment.

The catalytic stability of the photocatalyst was never ignored in the practical application of CO_2_ conversion. Figure 5a shows the cycling experiments of photocatalytic CO_2_ reduction for the optimal 30CBO/CuO sample. In the sixth cycling experiment, all the product yields were reduced by under 15%, and the CO selectivity still reached over 98.5%. The XRD and XPS results of the used sample after the sixth cycling experiment are shown in Figure 5b–d. In the XRD patterns (Figure 5b), no other new diffraction peaks of the used 30CBO/CuO sample were observed when compared with those of the fresh sample. Similarly, there was no obvious difference between the fresh and used samples in the XPS spectra (Figure 5c,d). It was indicated that the 30CBO/CuO composite exhibited an excellent photocatalytic stability for CO_2_ reduction with H_2_O vapor.

**Figure 6:**
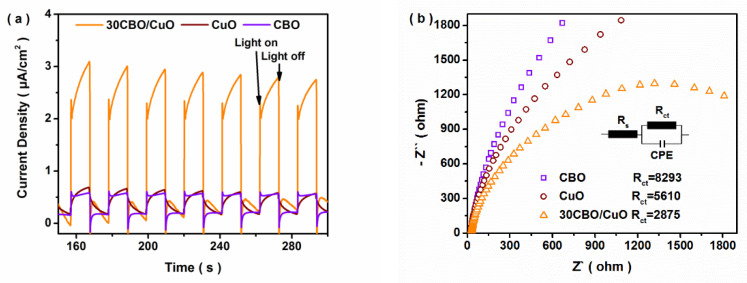
(**a**) I-t curves and (**b**) EIS Nyquist plots of CBO, CuO and 30CBO/CuO samples.

Transient photocurrent responses were recorded for several on-off irradiation cycles to provide more convincing evidence for the separation of photoinduced carriers. As shown in Figure 6a, the photocurrent responses of all the samples reproducibly increased under each irradiation and quickly recovered in the dark. Furthermore, the transient photocurrent of 30CBO/CuO was higher than those of the bare CBO and CuO samples, demonstrating that the composite exhibited a more efficient transfer and separation of photoinduced carriers [27]. Additionally, the EIS Nyquist plots are illustrated in Figure 6b. The semicircles in the high-frequency region correspond to the electron-transfer-limited process, which fit with the interfacial charge transfer resistance (R_ct_) [33]. The arc radius of 30CBO/CuO (R_ct_ 2875 Ω) was observed to be the smallest when compared with those of bare CBO (R_ct_ 8293 Ω) and CuO (R_ct_ 5610 Ω), indicating the most efficient charge transfer for the composite [11]. The above results proved the positive effect of the heterojunction on the carrier separation.

### 3.3. Possible Photocatalytic Mechanism

**Figure 7:**
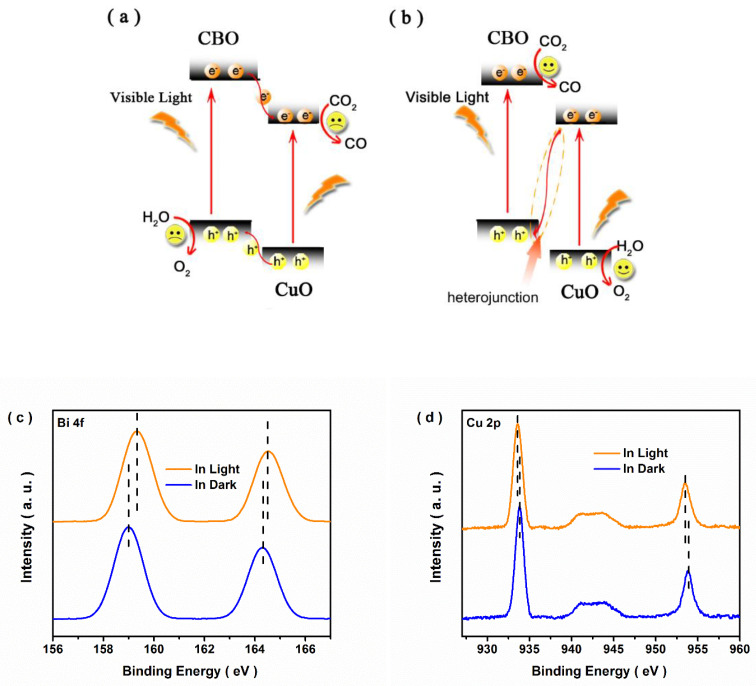
Schematic diagrams of the (**a**) common double-charge transfer and (**b**) S-scheme charge transfer modes in the CBO/CuO heterojunction; In situ-XPS spectra of (**c**) Bi 4f and (**d**) Cu 2p for the 30CBO/CuO sample.

Based on the above experimental results and the band energy alignment of the CBO/CuO heterojunction, the enhanced photocatalytic activity for CO_2_ reduction was deduced as follows. In the CBO/CuO composite, close contact between CBO and CuO caused the formation of a heterojunction, and an inner electrical field was established at the interface. Under visible-light illumination, the VB electrons of both components were excited. If the photoexcited charge carriers transferred following the common double-charge transfer mode (Figure 7a), the accumulated holes in the VB of the CBO component would not be able to oxidize H_2_O to O_2_ in thermodynamics, which was due to the VB potential of CBO (1.02 V vs. NHE) being more negative than the standard redox potential E^ө^(O_2_/H_2_O) (1.23 V vs. NHE) [33]. Meanwhile, no products were found when using the CBO sample in the photocatalytic experiment. If the S-scheme charge transfer mechanism mode in Figure 7b was employed, the catalytic sites of H_2_O oxidization were constructed on the surface of the CuO component. In thermodynamics, the holes on VB of CuO could realize O_2_ evolution from H_2_O molecules, and the experimental results also proved that the catalytic process of CO_2_ reduction and H_2_O oxidization were realized for the bare CuO sample [34]. In situ-XPS measurements of 30CBO/CuO were conducted for a further demonstration of electron transfer across the heterojunction interface. As shown in Figure 7c,d, two peaks of Bi 4f obviously shifted towards the lower binding energy direction under illumination, and two characteristic peaks of Cu 2p reversely shifted, implying that the photoinduced electrons transferred from CuBi_2_O_4_ to CuO [35,36]. Combined with the above analysis, the CBO/CuO composite was a direct S-scheme heterojunction photocatalyst.

Additionally, the conversion of CO_2_ to CH_4_ required eight electrons and four protons, while the conversion of CO_2_ to CO only required two electrons. CO formation was more favorable with a lower surface density of photogenerated electrons, and CH_4_ formation easily occurred with a higher surface density of photogenerated electrons [37,38]. Moreover, in the hydrogenation of CO_2_ and CO, H adatoms were obtained from H_2_O dissociation [37]. To explore the sample’s hydrogen production ability, the photocatalytic activity for water splitting was studied for the CBO/CuO composite. Unfortunately, no product was discovered when using the 30CBO/CuO sample. After Pt loading, the H_2_ yields for the Pt/CBO/CuO sample reached 12.4 μmol/g_cat_ after 8 h of illumination. Hence, the lack of aggregated electron sites and dissociation of H_2_O to H adatoms on the CuBi_2_O_4_ surface might cause the selectivity of CO formation for CO_2_ reduction in this study.

## 4. Conclusions

A series of CuBi_2_O_4_/CuO photocatalysts on glass substrates were synthesized by the ultra-fast spraying-calcination method. The optimal CBO/CuO composite exhibited a markedly higher photocatalytic performance of CO_2_ reduction with H_2_O vapor than those of bare CuBi_2_O_4_ and CuO, and the selectivity of the CO product was observably enhanced from below 18.5% to above 98.5%. After 9 h of visible-light illumination, the CO, CH_4_ and O_2_ yields reached 1599.1, 5.1 and 682.2 μmol/m^2^, respectively. In the sixth cycling experiment, the yields of the main products decreased by less than 15%, and a high CO selectivity was still kept. The enhanced activity of CO_2_ reduction was attributed to the efficient separation of photogenerated charge carriers that originated from the well-aligned staggered band structure of the CuBi_2_O_4_/CuO heterojunction. Based on the photocatalytic activity and in situ-XPS results, the S-scheme charge transfer mechanism was finally proposed. In summary, this study is expected to be useful in developing S-scheme photocatalysts for CO_2_ reduction and to provide some meaningful information for a deep understanding of how photoinduced electrons transfer across contact interfaces.

## Data Availability

Data are contained within the article.

## Supplementary Materials

The following supporting information can be downloaded at: https://www.mdpi.com/article/10.3390/nano12183247/s1, Table S1: Binding energy and band gap of the obtained samples by XPS and DRS measurements; Figure S1: Survey spectra (a) and high resolution O 1s XPS spectra (b) of the CBO, CuO and 30CBO/CuO samples; Figure S2: TEM image of CBO (a) and CuO (b) samples (inset: diameter size distribution); Figure S3: Photocatalytic activity for different samples after 3 h of visible-light illumination.
