# Supplementary material for: Ultra-Fast Construction of Novel S-Scheme CuBi2O4/CuO Heterojunction for Selectively Photocatalytic CO2 Conversion to CO
Source: Nanomaterials (Basel). 2022 Sep 19;12(18):3247. doi: 10.3390/nano12183247 (PMC9504175; doi:10.3390/nano12183247)
Supplement: Supplementary file 1 [file nanomaterials-12-03247-s001.zip › nanomaterials-1894714-supplementary.pdf]

Supplementary Material

Ultra-Fast Construction of Novel S-Scheme $CuBi_2O_4/CuO$ Heterojunction for Selectively Photocatalytic CO_2 Conversion to CO

**Weina Shi [1], Xiu Qiao [2], Jichao Wang [2,*], Miao Zhao [1], Hongling Ge [2], Jingjing Ma [2], Shanqin Liu [2]
and Wanqing Zhang [2,*]**

1 School of Chemistry and Materials Engineering, Xinxiang University, Xinxiang 453000, China
2 College of Chemistry and Chemical Engineering, Henan Institute of Science and Technology, Xinxiang 453000, China
* Correspondence: wangjichao@hist.edu.cn (J.-C.W.); zhangwqzzu@163.com (W.Z.); Tel.: +86-0373-304-0418 (J.-C.W.& W.Z.)

Table S1. Binding energy and band gap of the obtained samples by XPS and DRS measurements

Sample	Bi 4f (eV)	Cu 2p (eV)	VB (eV)	Eg (eV)
CBO	158.84	-	1.02	1.86
CuO	-	934.01	1.44	1.70
30CBO/CuO	158.95	933.84	-	-

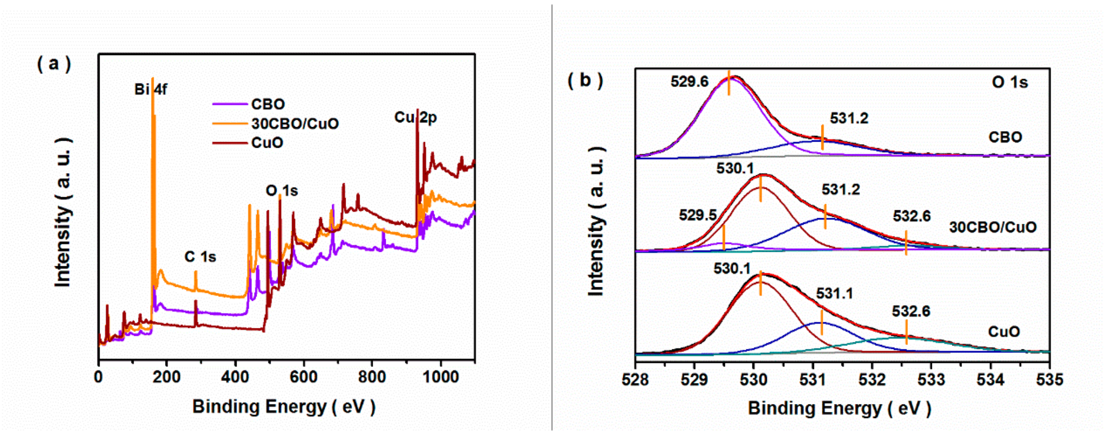

Figure S1. Survey spectra (a) and high resolution O 1s XPS spectra (b) of the CBO, CuO and 30CBO/CuO samples.

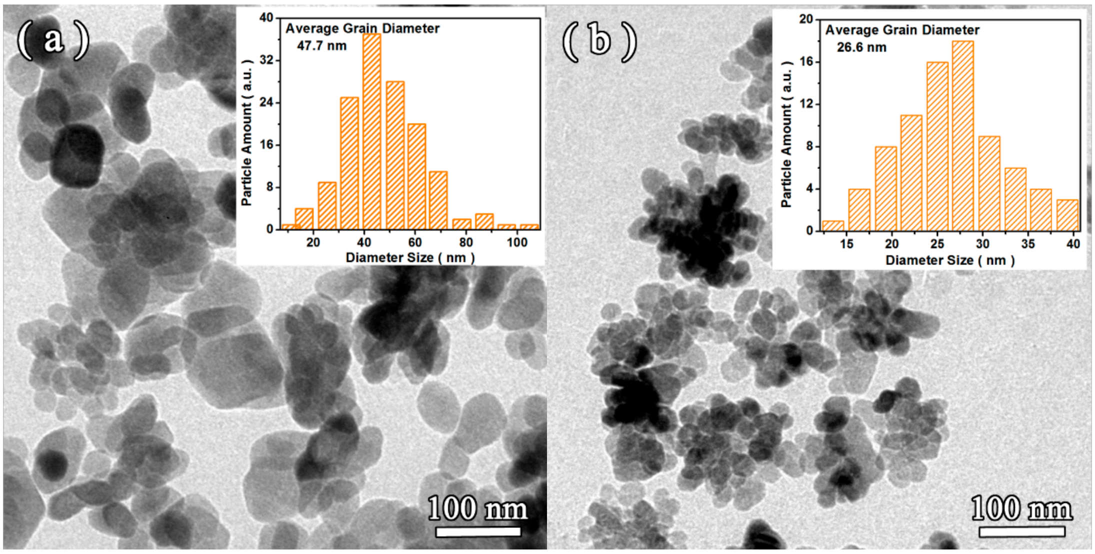

Figure S2. TEM image of CBO (a) and CuO (b) samples (inset: diameter size distribution)

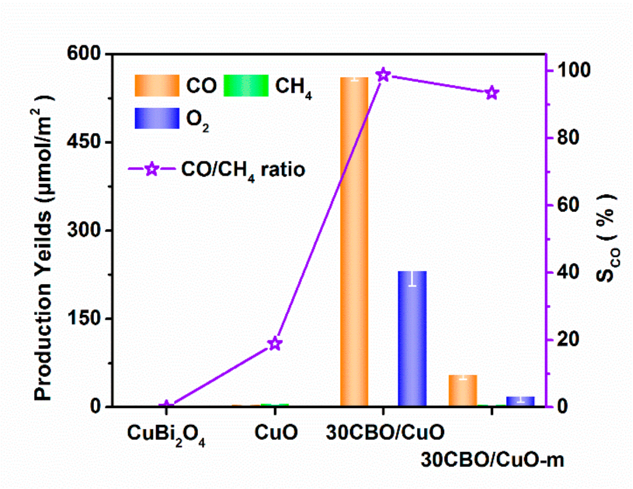

Figure S3. Photocatalytic activity for different samples after 3 h of visible-light illumination.
